# Synthesis method of asymmetric gold particles

**DOI:** 10.1038/s41598-017-02485-7

**Published:** 2017-06-07

**Authors:** Bong-Hyun Jun, Michael Murata, Eunil Hahm, Luke P. Lee

**Affiliations:** 1 Department of Bioscience and Biotechnology, Konkuk University, Seoul, 143-701 Republic of Korea; 2 Department of Bioengineering, Biomolecular Nanotechnology Center, Berkeley Sensor and Actuator Center, University of California, Berkeley, California, 94720 United States

## Abstract

Asymmetric particles can exhibit unique properties. However, reported synthesis methods for asymmetric particles hinder their application because these methods have a limited scale and lack the ability to afford particles of varied shapes. Herein, we report a novel synthetic method which has the potential to produce large quantities of asymmetric particles. Asymmetric rose-shaped gold particles were fabricated as a proof of concept experiment. First, silica nanoparticles (NPs) were bound to a hydrophobic micro-sized polymer containing 2-chlorotritylchloride linkers (2-CTC resin). Then, half-planar gold particles with rose-shaped and polyhedral structures were prepared on the silica particles on the 2-CTC resin. Particle size was controlled by the concentration of the gold source. The asymmetric particles were easily cleaved from the resin without aggregation. We confirmed that gold was grown on the silica NPs. This facile method for synthesizing asymmetric particles has great potential for materials science.

## Introduction

Asymmetric particles have drawn considerable attention in recent years for their novel properties. Owing to their unique intra-particle potential for coupling and local field enhancement, applications include the fabrication of optical, optoelectronic, and sensing devices such as for targeted cellular imaging systems^1–5^. Therefore, the size and shape of particles are critical factors in determining their material properties. Thus, the ability to control these parameters throughout the synthesis process has become a major goal in the field of materials science^6–12^. So far, synthesis techniques have been reported for a few other low symmetry metal particles including nano-rods and nano-clusters, but research into the scale-up fabrication of asymmetric particles can be further developed in some structures^13^. These synthesis methods utilize kinetic control over nucleation of the nano-cluster through a carefully determined polymer concentration for steric stabilization, which limits their ability to scale up due to aggregation. E-beam methods combined with 2-D plate were reported^4,5,14^. However, these methods only use a small area due to the limitation of the e-beam method and 2-D plate size, quantities of particles can be highly limited. A facile and widely applicable method for synthesizing a variety of asymmetric particles in large quantities would help exploit their potential and pave the way for a new field in asymmetric-particle-based science.

Herein, we report a novel method for the preparation of various asymmetric particles. In our approach, micro-sized spherical beads were used as capture templates for nano-sized silica spheres. Then, asymmetric gold particles were grown on the nanometer silica spheres. Different size and shape of gold particles could be grown by changing the concentration of the metal source and the type of solvent. The gold structures can be obtained as particles.

## Results and Discussion

**Figure 1:**
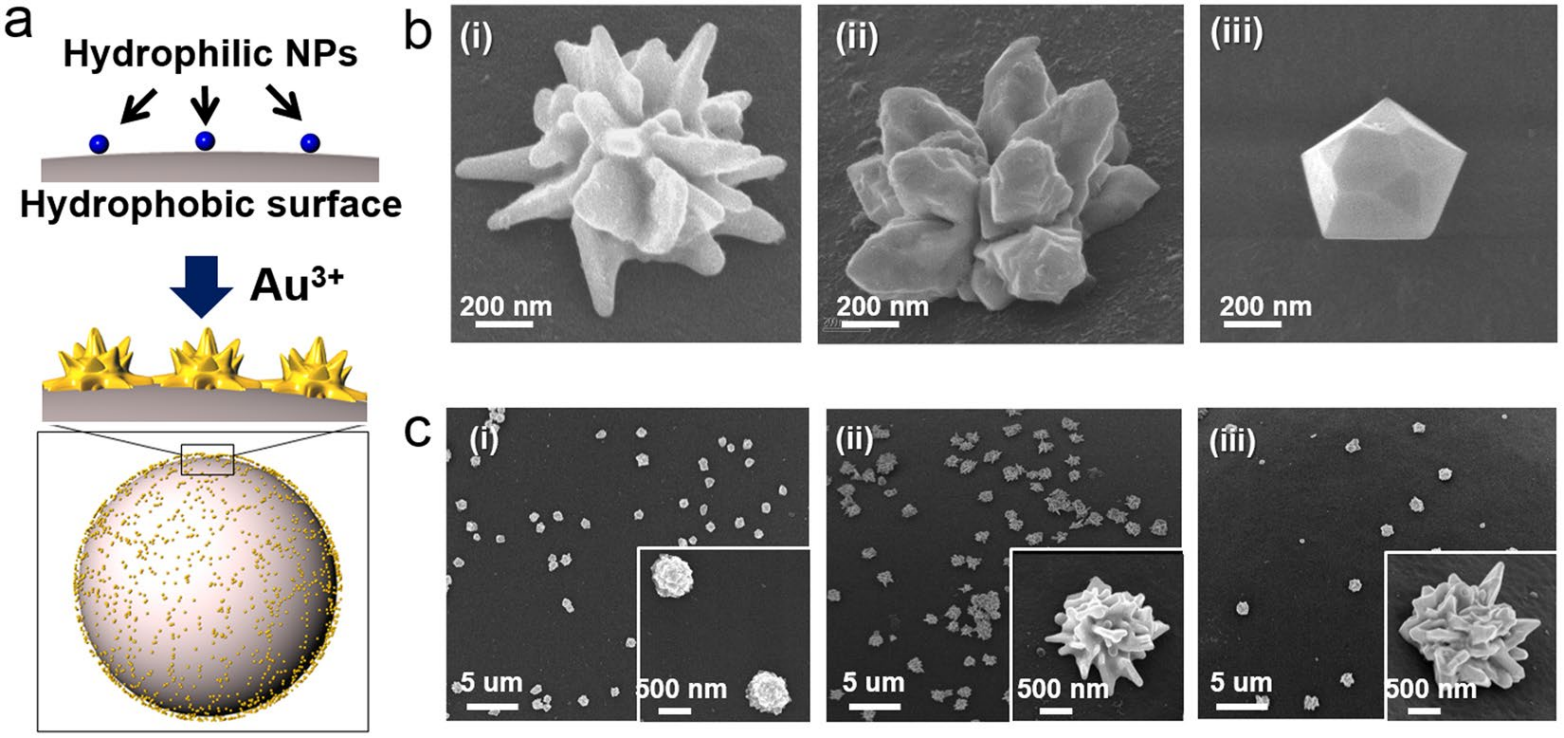
Cleavage from the beads and half-planar particles. (**a**) Illustration of beads and NPs, (**b**) SEM image of bead, (**c**) high magnification SEM image of bead, (**d**) low magnification SEM image of asymmetric nanorose, (**e**) side view of nanorose (**f**) top view of nanorose particle.

The fabrication method for asymmetric particles is illustrated in Fig. 1. Two types of backbones were used: a micro-sized (72–150 µm) immobilized spherical polymer with 2-chloritylchloride linkers (2-CTC resin) and nano-sized silica spheres (120 nm, see supporting Fig. 1).

2-CTC resins are widely used for solid-phase peptide synthesis. Their key advantages are lower cost and recyclability^15–17^. The 2-CTC resins can form a covalent bond with nucleophilic functional groups such as thiols^16^, amines^18^, and carboxyls^19^. This bond can be cleaved easily under mildly acidic conditions. (see supporting Fig. 2) To immobilize silica nanoparticles (NPs) onto the 2-CTC resin, thiol-functionalized 90 nm silica NPs were prepared^20^ and mixed with 2-CTC resin under basic conditions. Here, we used dimethyl sulfoxide (DMSO) as the solvent, which is aprotic and polar. Because protic solvents can compete with the nucleophilic substitution reaction of the 2-CTC group, an aprotic solvent was used. Among aprotic solvents, polar solvents are compatible with the hydrophilic functionalized silica NPs and incompatible with the hydrophobic resin, causing shrinkage of the resin and preventing the trapping of NPs inside the resin. After the reaction, the remaining silica NPs and excess reagents and solvents were removed from the silica NP immobilized resin by filtration and washed with ethanol. The silica NPs immobilized on the 2-CTC resin were analyzed by scanning electron microscopy (SEM). Silica NPs were successfully immobilized onto the surface of 2-CTC resin (see supporting Fig. 3b). Various silica NPs such as amine-functionalized NPs (50 and 120 nm) and thiol-functionalized NPs (200 nm) were also immobilized (see supporting Fig. 3c–e). The silica NP loading amounts can be controlled by tuning their concentration (Data not shown).

**Figure 2:**
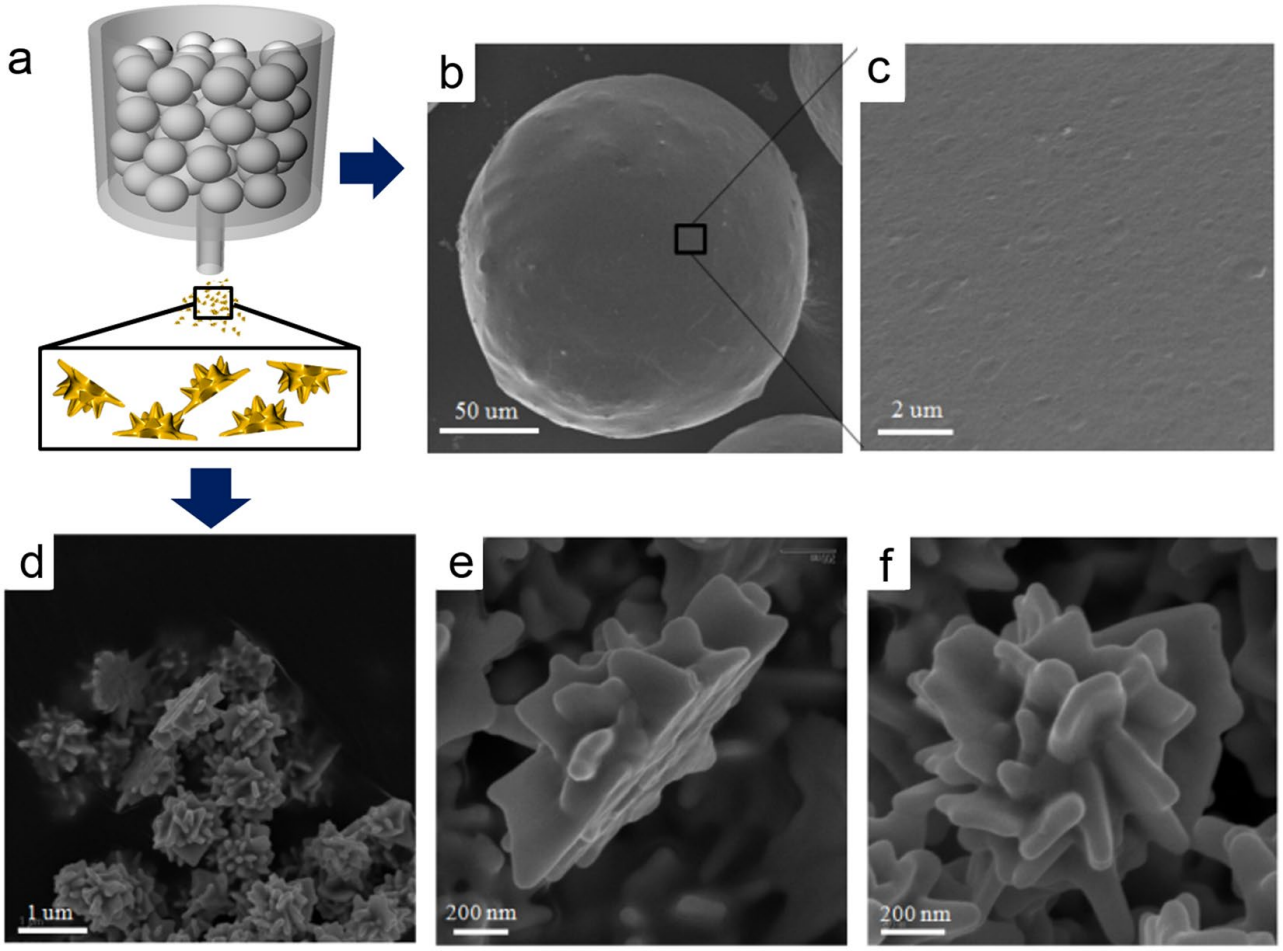
Schematic illustration of the synthesis of half-planar gold particles. (**a**) 2-CTC resin, (**b**) silica NPs immobilized resin, (**c**) gold NPs immobilized on the resin, (**d**) cleavage of asymmetric gold NPs from resin, (**e**) filtration to obtain the asymmetric gold NPs; 2-CTC resin remained in the filter, and (**f**) obtained asymmetric gold NPs.

The gold source (1% w/w in DI water) and reductant (hydroxylamine, 0.5 mg/mL in water) were then added for preparing asymmetric particles. One exclusive advantage of our method comes from combining a hydrophobic resin with hydrophilic silica NPs. Because hydrophilic gold sources in H_2_O prefer hydrophilic surfaces rather than hydrophobic surfaces, gold is able to grow on the silica NPs under certain conditions, as shown in Fig. 2a. Moreover, the silica NPs form strong covalent bonds with the beads, permitting the application of various conditions without concern for stability. Here, we altered the gold ion concentration and solvent. As a result, we could synthesize gold NPs with a rose or polyhedral shape on the beads as shown in Fig. 2b. (see supporting Fig. 4) When the concentration of gold was increased, the average size of the particles also increased, as shown in Fig. 2c (see supporting Fig. 5).

Generally, particles aggregate without charge repulsion or spacers to reduce surface energy in the synthesis step. This is one of the most critical considerations in the synthesis of colloidal particles, as shown in supporting Fig. 6. Because the silica NPs were physically separated from each other in our method, the particles which have gap between particles did not aggregate during the gold growth step. (see supporting Fig. 7) Moreover, target NPs were immobilized on the larger micro-sized resin such that even if nucleation were to happen, the nucleated NPs could easily be removed from larger micro-sized resin by filtration. Thus, nucleation of gold in solution was not a concern. These advantages enabled us to apply a variety of conditions for gold growth.

Among the various shapes shown in Fig. 2b, the rose-shaped particle on the beads (Fig. 2bi) was the model for this study. Before the particles were cleaved from the beads, mercaptopropionic acid, which has a thiol group on one side and a carboxylic group on the other side, was added to generate charge repulsion and prevent aggregation.

To obtain the synthesized particles from the resin, the bonds were cleaved using mildly acidic conditions, 1–2% trifluoroacetic acid (TFA) in methylene chloride in separate reaction vessels (i.e., a Libra tube with a filter).

**Figure 3:**
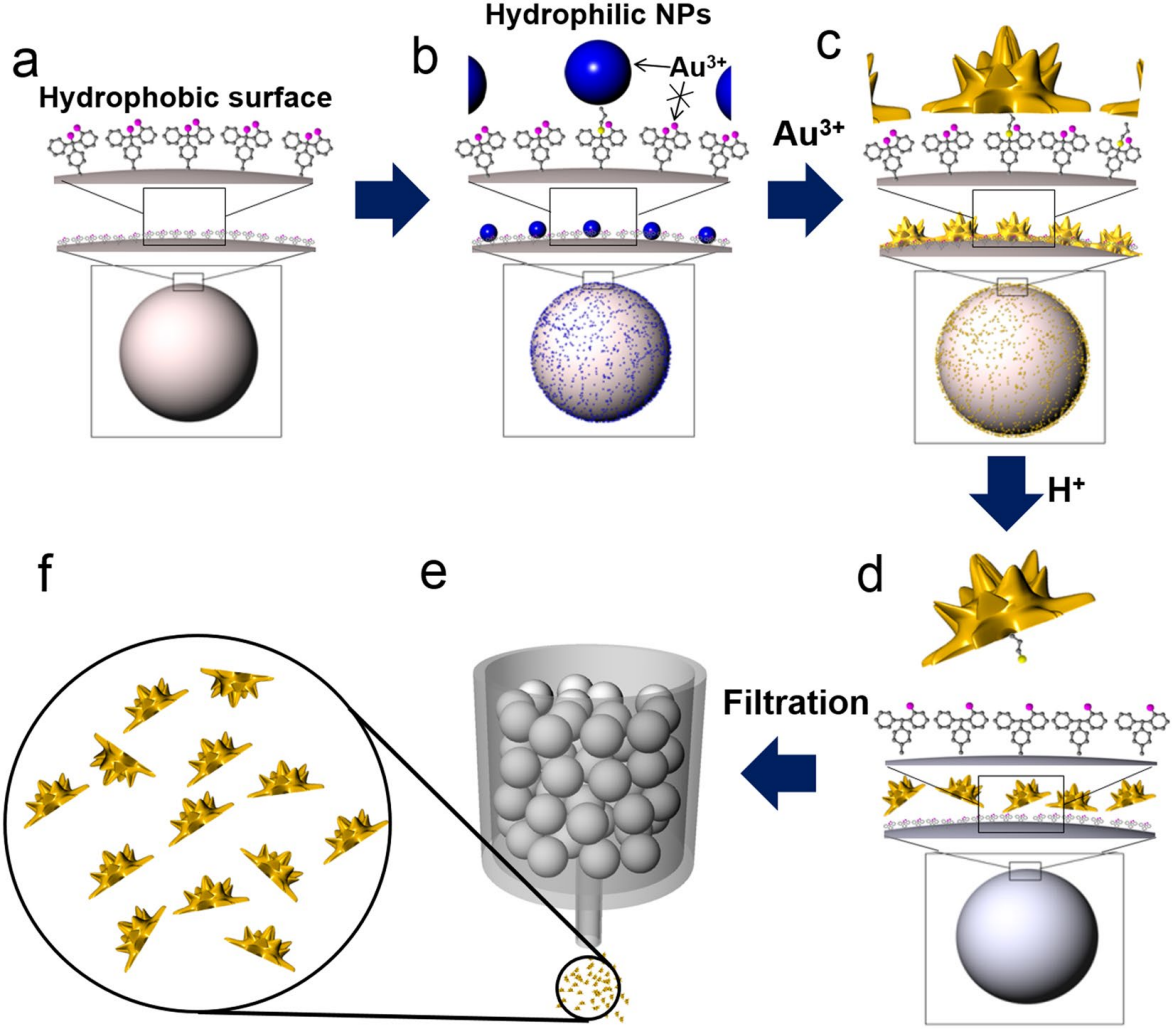
Gold NPs of various shapes and sizes immobilized on the beads. (**a**) Illustration of gold growth on the silica NPs. (**b**) SEM images of gold coated silica NPs on the beads (200 µM) (i) in H_2_O solvent (stirring), (ii) in H_2_O solvent (shaking) (iii) in EtOH solvent, (**c**) SEM images of gold coated silica NPs on the beads (in H_2_O solvent) (i) 50 µM, (ii) 200 µM, (iii) 800 µM.

This cleavage step is a well-known chemical reaction^20,21^ that results in the 2-CTC groups remaining on the resin (>72 µm) and a mixture containing the asymmetric NPs (<1 µm), as shown in Fig. 3a. The materials in the mixture were separated by filtration, and each material was analyzed by SEM. As shown in Fig. 3b–c, the resin surface was clean, which implies that the asymmetric particles were completely cleaved from the 2-CTC resin. We believe that the half-planar structure is formed on the silica NPs on the microsized beads because one side of the silica NP is physically blocked by the resin, and gold can only grow on the exposed side. The cleavage step does not affect particle size, morphology or shape, and thus, we were able to obtain particles with a rose shape similar to the ones on the beads: planar on one side and rose-shaped on the other, as shown in Fig. 3d–f.

In this study, we synthesized nanorose particles on a 10-mg-scale; however, the procedure has a potential to be scaled up for use in large-scale synthesis (see supporting Fig. 8 and info 1). The particles were further analyzed by transmission electron microscopy (TEM) (see supporting Fig. 9a) and dark field microscopy. When the nanorose particles were irradiated by the TEM electron beam (80 kV), the shape changed from rose to spherical (see supporting Fig. 9b). Additionally, silica from the NPs that were used as the template for the asymmetric synthesis vacated the cavity within the nanorose particles, which implies that the synthesized rose particles contained silica NPs (see supporting Figs 9c and 10). The particles exhibited multiple scattering in dark field spectrum. (see supporting Fig. 11)

We report a method for the synthesis of gold asymmetric particles. Silica NPs were successfully immobilized onto a hydrophobic microsized 2-CTC resin. Then, gold grew on the silica NPs attached to the beads, and the various nanostructures were prepared. The colloidal particles were obtained by cleavage from the resin and the obtained particles exhibited asymmetric structures. The method has potential for the fabrication of not only half-planar half-rose structures as demonstrated, but also various other asymmetric structures, including asymmetric structures based on other metals such as Ag, Cu and Pd, for example. Furthermore, these asymmetric particles also have the potential for selective functionalization, resulting from the exposed linker that bound the silica nanoparticle to the 2-CTC resin. This method could be a facile and widely applicable method for synthesizing a variety of asymmetric particles in larger quantities than would be accessible with alternative techniques such as e-beam lithography; thus, it could have great potential in asymmetric-particle-based science.

## Method

### Preparation of thiol functionalized silica nanoparticles (NPs)

Silica NPs were prepared using the well-known Stober method. A 1.6 mL portion of tetraethyl orthosilicate (TEOS) was added to 40 mL of ethanol. Then, 3 mL of ammonium hydroxide was added to the ethanol solution under vigorous magnetic stirring (320 rpm). The resulting mixture was stirred for 24 h at room temperature. The resulting silica NPs were centrifuged and washed with ethanol three times. The size of the prepared NPs was ~120 nm, as shown in Supporting Fig. 1. These silica NPs were then thiol functionalized using 60 μL of 3-mercaptopropyltrimethoxysilane (MPTS) and 100 uL of ammonium hydroxide, which were added to 5 mL of the dispersed silica NP solution (20 mg/mL in ethanol) and stirred for 12 h at room temperature. The thiol-functionalized silica NPs were centrifuged and washed with ethanol three times and with DMSO three times.

### Preparation of half-planar gold particles

First, the thiol-functionalized silica NPs were immobilized on the 2-CTC resin (Beadtech, Inc., Korea) using a standard peptide synthesis method. A 2 mL portion of the silica NP solution (50 mg/mL in DMSO) was added to 2 g of 2-CTC resin beads in reaction vessels. Then, 200 μL of *N*,*N*-diisopropylethylamine (DIPEA) was added to the DMSO solution and shaken for 12 h at room temperature. A 40 μL to 3 mL volume of gold source (1% w/w in DI water) was added to thiol-modified silica immobilized beads (50 mg) in water under vigorous magnetic stirring. Then, 500 μL of hydroxylamine (0.5 mg/mL in water) was added. After the reaction (typically overnight), the remaining reagents were washed with water and ethanol, and the reaction vessel was filled with 10 mL of ethanol. Then, the surface of the gold particles on the beads in solution was coated with mercaptocarboxylic acid, which contains a thiol group, so that a negative surface charge is produced to prevent aggregation. This was accomplished by first washing with ethanol, then with methylene chloride, and adding 100 μL of mercaptocarboxylic acid to the beads and shaking for 1 h. Finally, the particles were cleaved from the beads in a mildly acidic environment with 2 mL TFA for 1 h. The solution was collected by filtration (impurities from the 2-CTC resin could also be detached from beads. However, they were removed in our experiments). The particles were obtained by centrifugation^18–22^.

## Electronic supplementary material

Supplementary info
