# Publisher Correction: Synthesis method of asymmetric gold particles

**DOI:** 10.1038/s41598-017-14993-7

**Published:** 2017-11-21

**Authors:** Bong-Hyun Jun, Michael Murata, Eunil Hahm, Luke P. Lee

**Affiliations:** 1 Department of Bioscience and Biotechnology, Konkuk University, Seoul, 143-701 Republic of Korea; 2 Department of Bioengineering, Biomolecular Nanotechnology Center, Berkeley Sensor and Actuator Center, University of California, Berkeley, California, 94720 United States

*Scientific Reports* **7**:2921; doi:10.1038/s41598-017-02485-7; Article published online 07 June 2017

This Article contains an error in the order of the Figures and Figure legends. In the HTML version Figures 1, 2 and 3 were published as Figures 3, 1 and 2 respectively. In the PDF version the Figures 1, 2 and 3 were published as Figures 2, 3 and 1 respectively.

The correct Figures 1, 2 and 3 appear below along with their accompanying legends.

**Figure 1:**
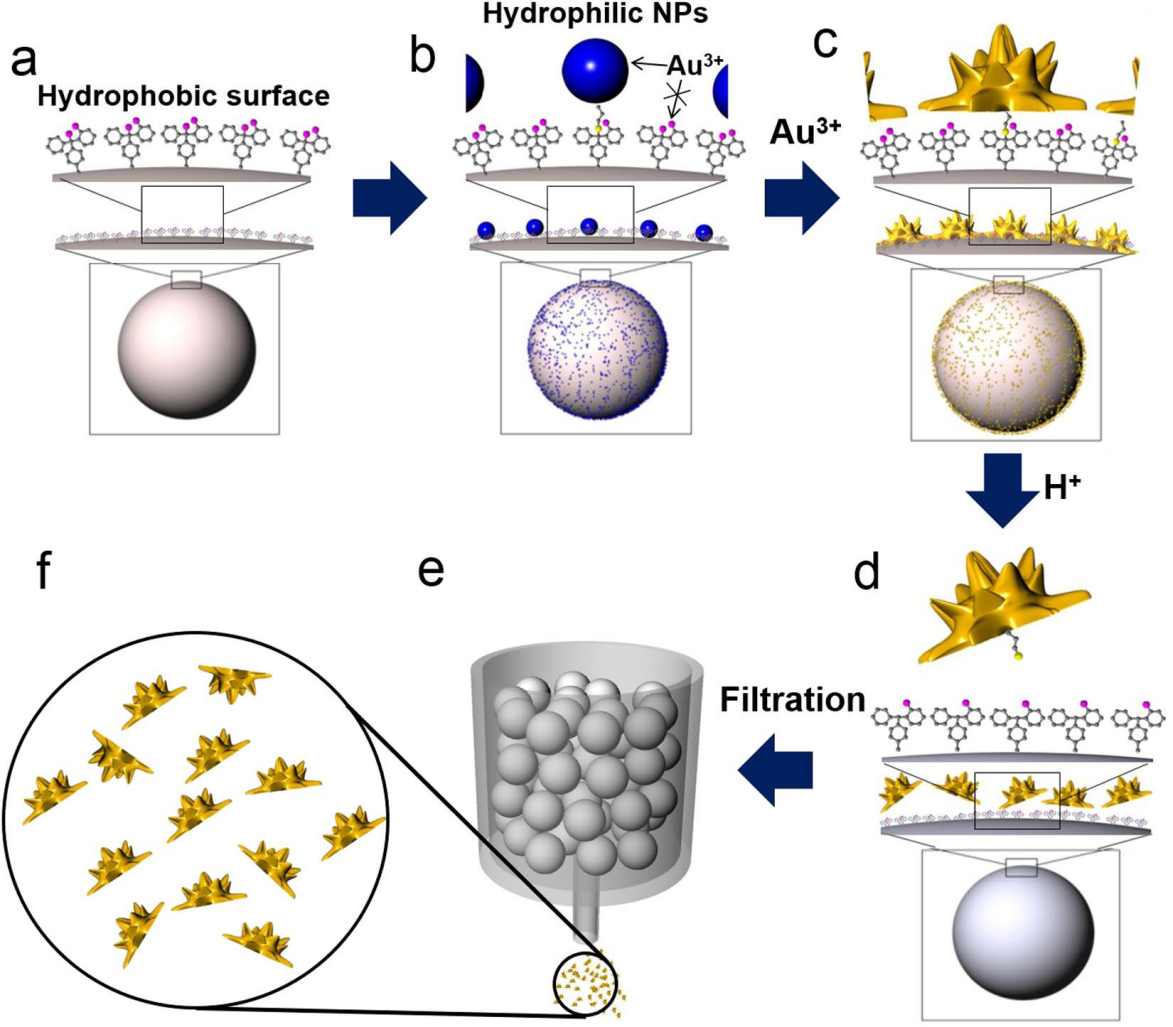
Schematic illustration of the synthesis of half-planar gold particles. (**a**) 2-CTC resin, (**b**) silica NPs immobilized resin, (**c**) gold NPs immobilized on the resin, (**d**) cleavage of asymmetric gold NPs from resin, (**e**) filtration to obtain the asymmetric gold NPs; 2-CTC resin remained in the filter, and (**f**) obtained asymmetric gold NPs.

**Figure 2:**
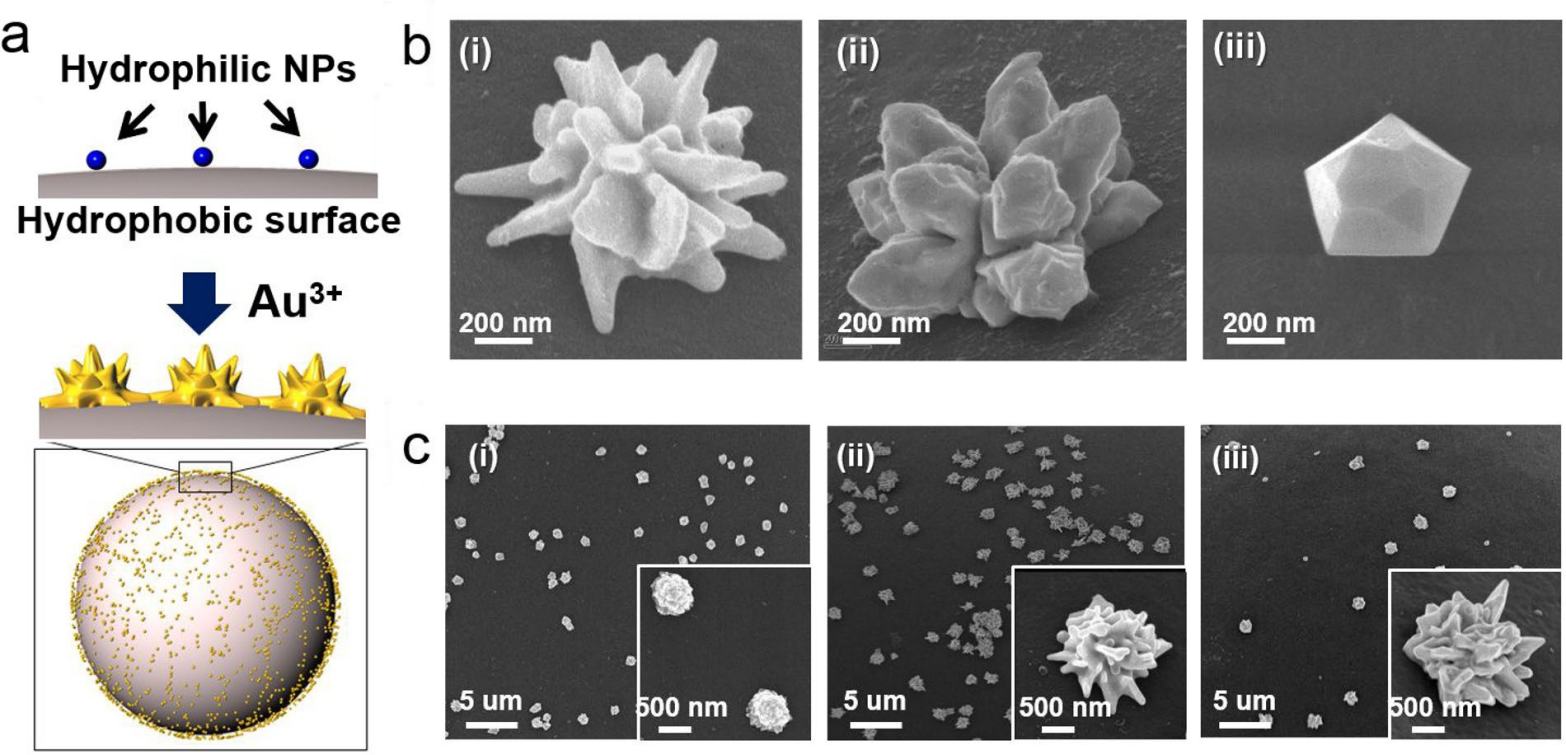
Gold NPs of various shapes and sizes immobilized on the beads. (**a**) Illustration of gold growth on the silica NPs. (**b**) SEM images of gold coated silica NPs on the beads (200 µM) (i) in H2O solvent (stirring), (ii) in H2O solvent (shaking) (iii) in EtOH solvent, (**c**) SEM images of gold coated silica NPs on the beads (in H2O solvent) (i) 50 µM, (ii) 200 µM, (iii) 800 µM.

**Figure 3:**
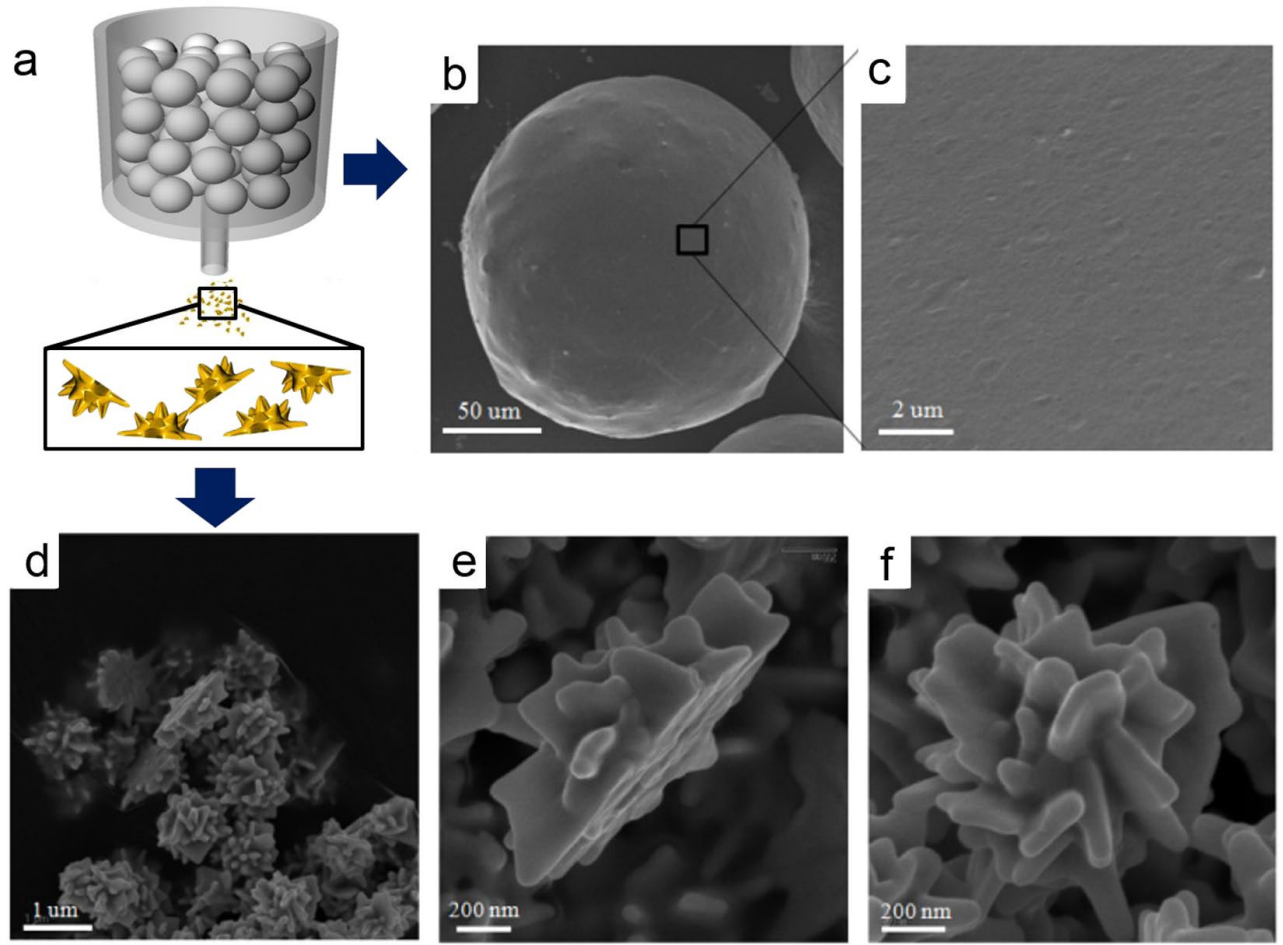
Cleavage from the beads and half-planar particles. (**a**) Illustration of beads and NPs, (**b**) SEM image of bead, (**c**) high magnification SEM image of bead, (**d**) low magnification SEM image of asymmetric nanorose, (**e**) side view of nanorose (**f**) top view of nanorose particle.

