# Supplementary material for: Synthesis method of asymmetric gold particles
Source: Sci Rep. 2017 Jun 7;7:2921. doi: 10.1038/s41598-017-02485-7 (PMC5462826; doi:10.1038/s41598-017-02485-7)
Supplement: Supplementary file 1 — Supplementary info [file 41598_2017_2485_MOESM1_ESM.doc]

**Supporting Information**

Synthesis method of asymmetric gold particles

Bong-Hyun Jun,^a,b*^ Michael Murata,^a^ Eunil Hahm,^b^ and Luke P. Lee^a,*^

a Department of Bioengineering, Biomolecular Nanotechnology Center, Berkeley Sensor and Actuator Center, University of California, Berkeley, California 94720, United States.

b Department of Bioscience and Biotechnology, Konkuk university, Seoul 143-701, Republic of Korea

[*bjun@konkuk.ac.kr](mailto:*bjun@konkuk.ac.kr), * lplee@berkeley.edu


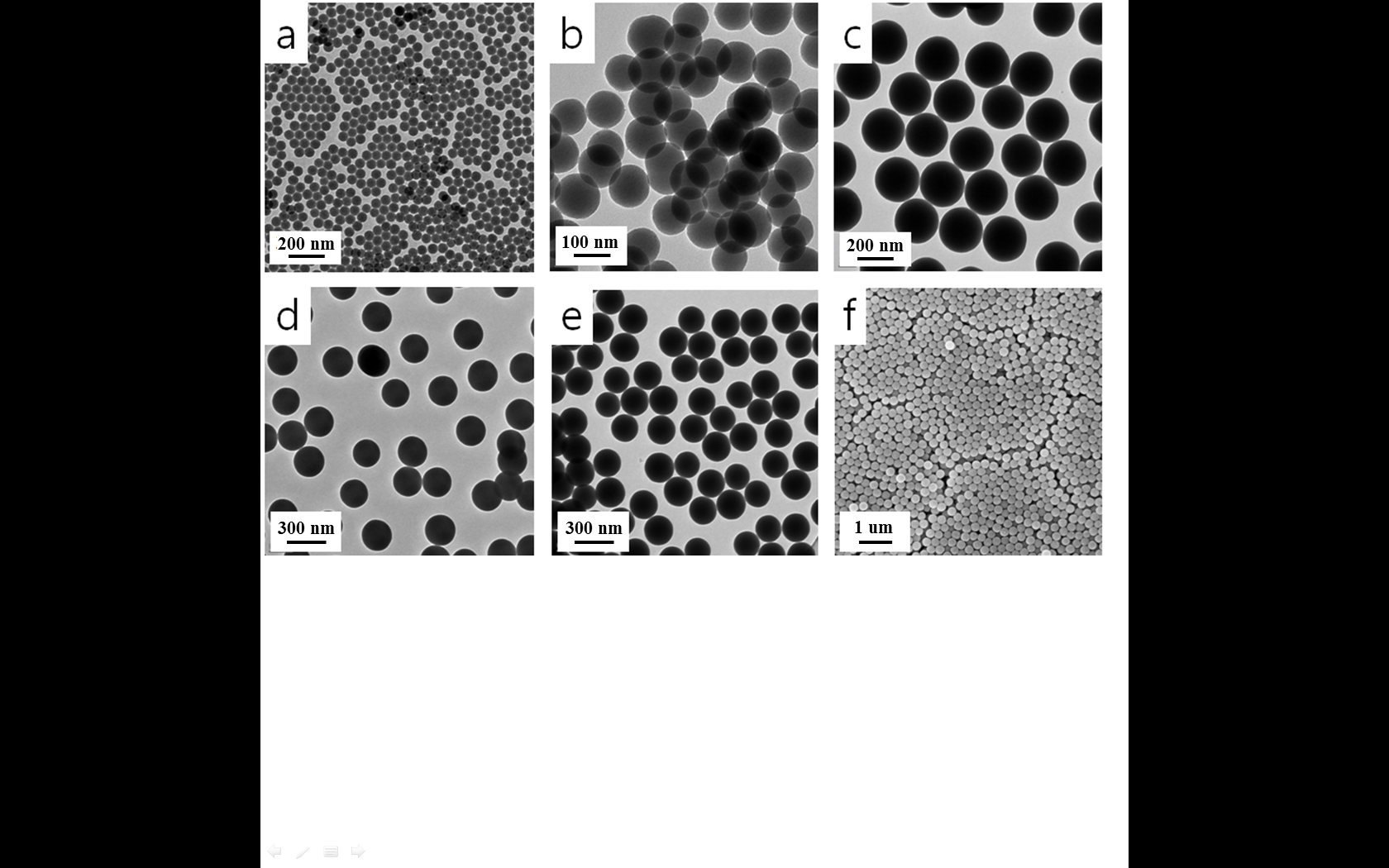


Supporting Figure 1. Electron microscope images of silica NPs. a) TEM image of 50 nm silica NPs, b) 120 nm silica NPs, c) 230 nm silica NPs, d) thiol functionalized 230 nm silica NPs, e) amine functionalized 230 nm silica NPs, and f) SEM images of thiol functionalized 230 nm silica NPs.


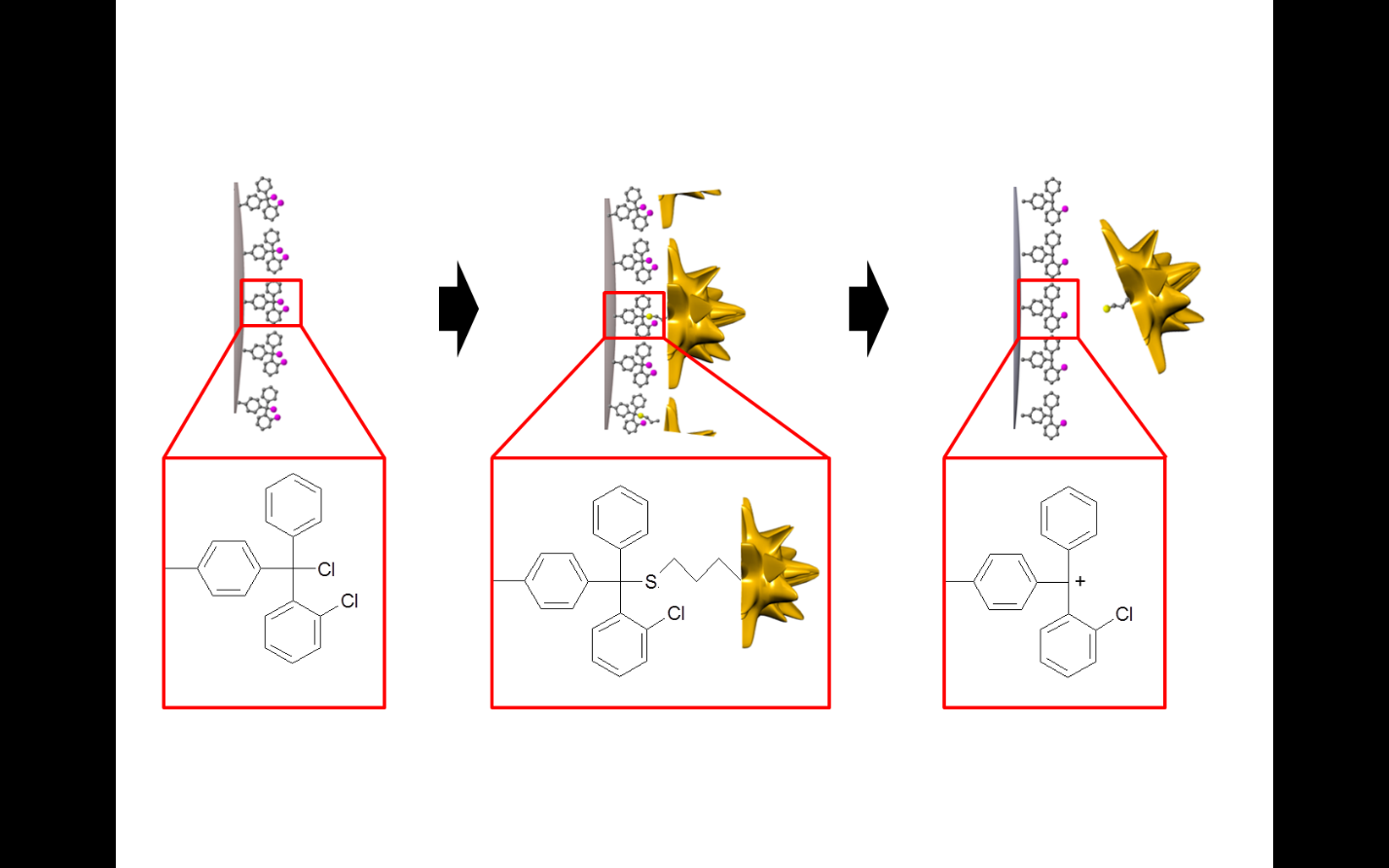


Supporting Figure 2. Illustration of binding and cleaving of thiol-functionalized particle to 2-CTC group


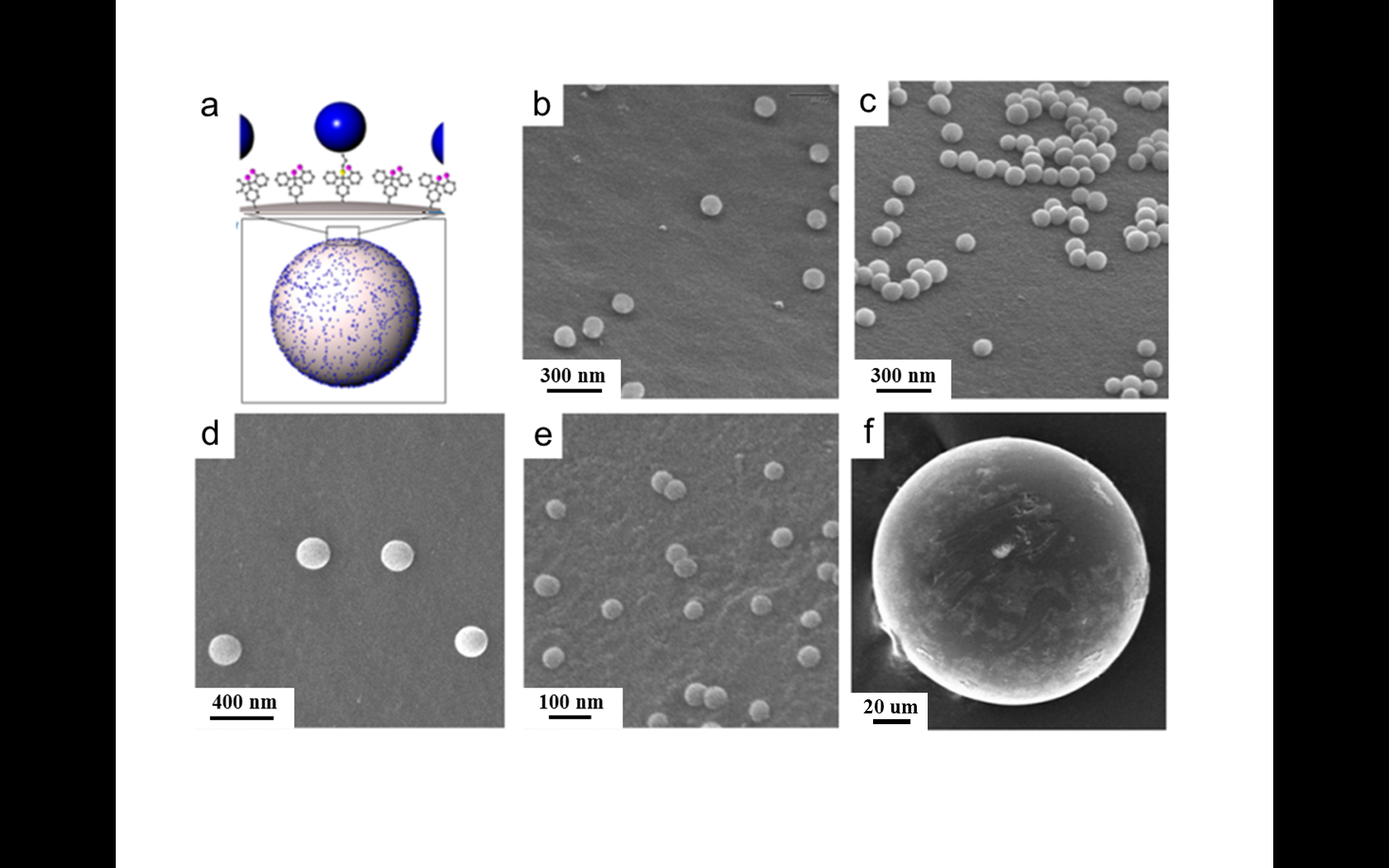


Supporting Figure 3. Silica NPs immobilized on the beads. a) Illustration of silica NPs immobilized on the beads, b) SEM image of thiol-functionalized 120 nm silica NPs on the beads, c) SEM image of amine-functionalized 120 nm silica NPs on the beads, d) SEM image of thiol-functionalized 200 nm silica NPs on the beads, e) SEM image of thiol-functionalized 50 nm magnetic silica NPs on beads, f) SEM image of the beads with immobilized 120 nm silica NPs.


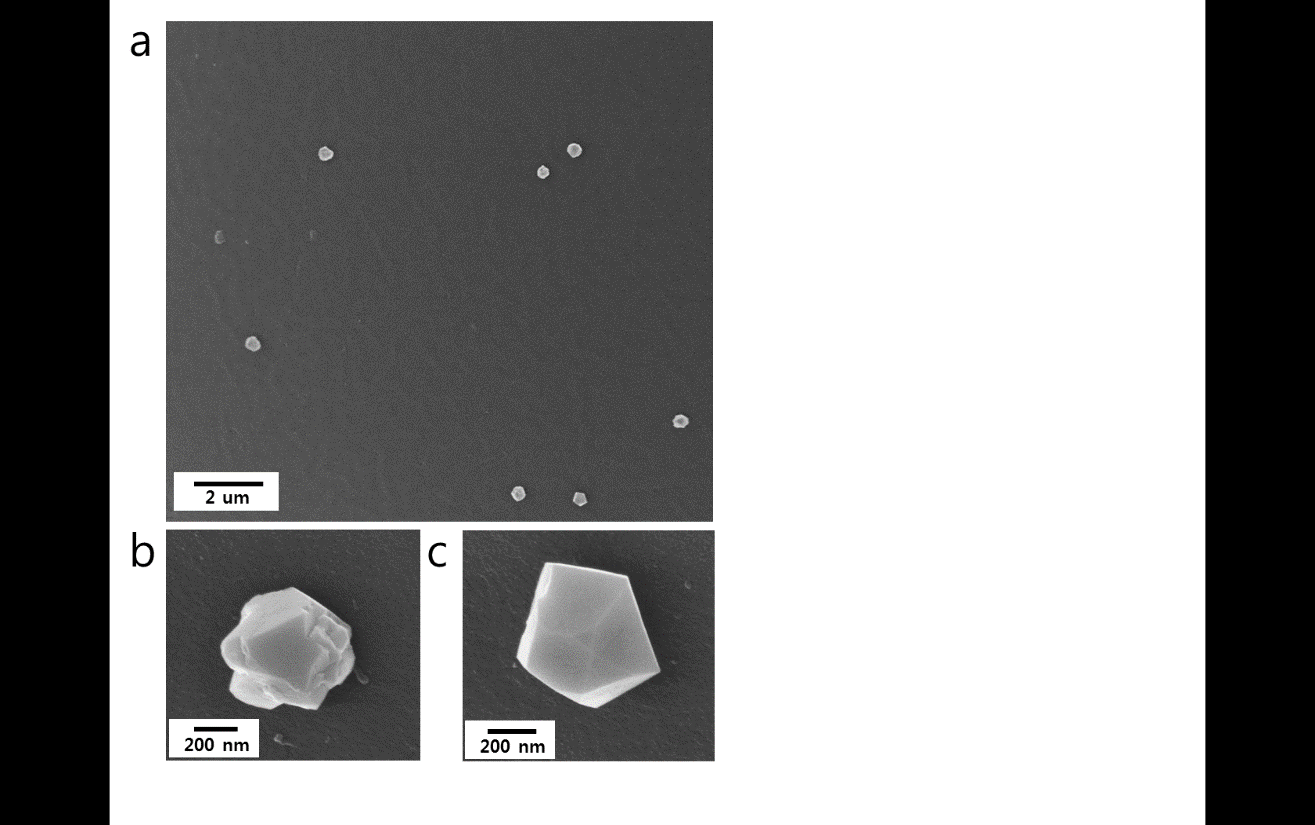


Supporting Figure 4. SEM images of Fig2biii) (in EtOH solvent condition). a) Low magnification, b-c) high magnification

The solvent can affect the gold growth to form different structures depending on the solvent. One of potential reasons for the difference in gold particle shape could be that the bulkiness of ethanol relative to that of water allows water to increase interfacial rigidity whereas ethanol creates a more fluid interface. A lower surface energy permits the growth of gold in all directions while high interfacial energies facilitates growth along initial nucleation paths.^2-3^ Thus, the difference in surface rigidity causes ethanol to produce polyhedral shapes whereas water creates the nano-rose structure.


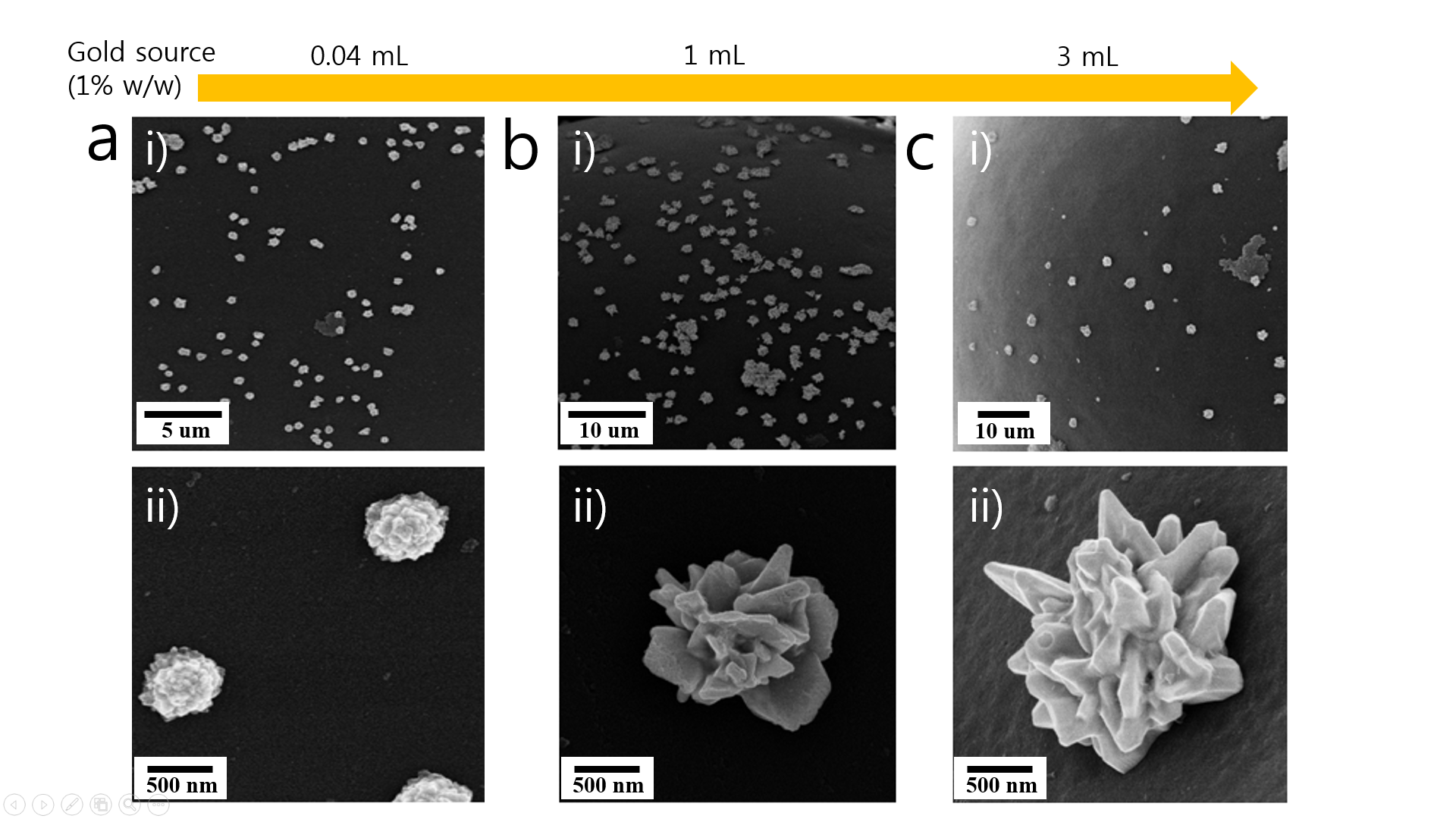


Supporting Figure 5. a) 50 uL Au source b) 1 mL Au source c) 3 mL Au source with same condition with Fig2bi. i) Low magnification, ii) high magnification

When Au source (1% w/w) amount was increased (50 µL, 1 mL and 3 mL), the size of particles was increased (497±100, 1,328±341 and 1605±402).


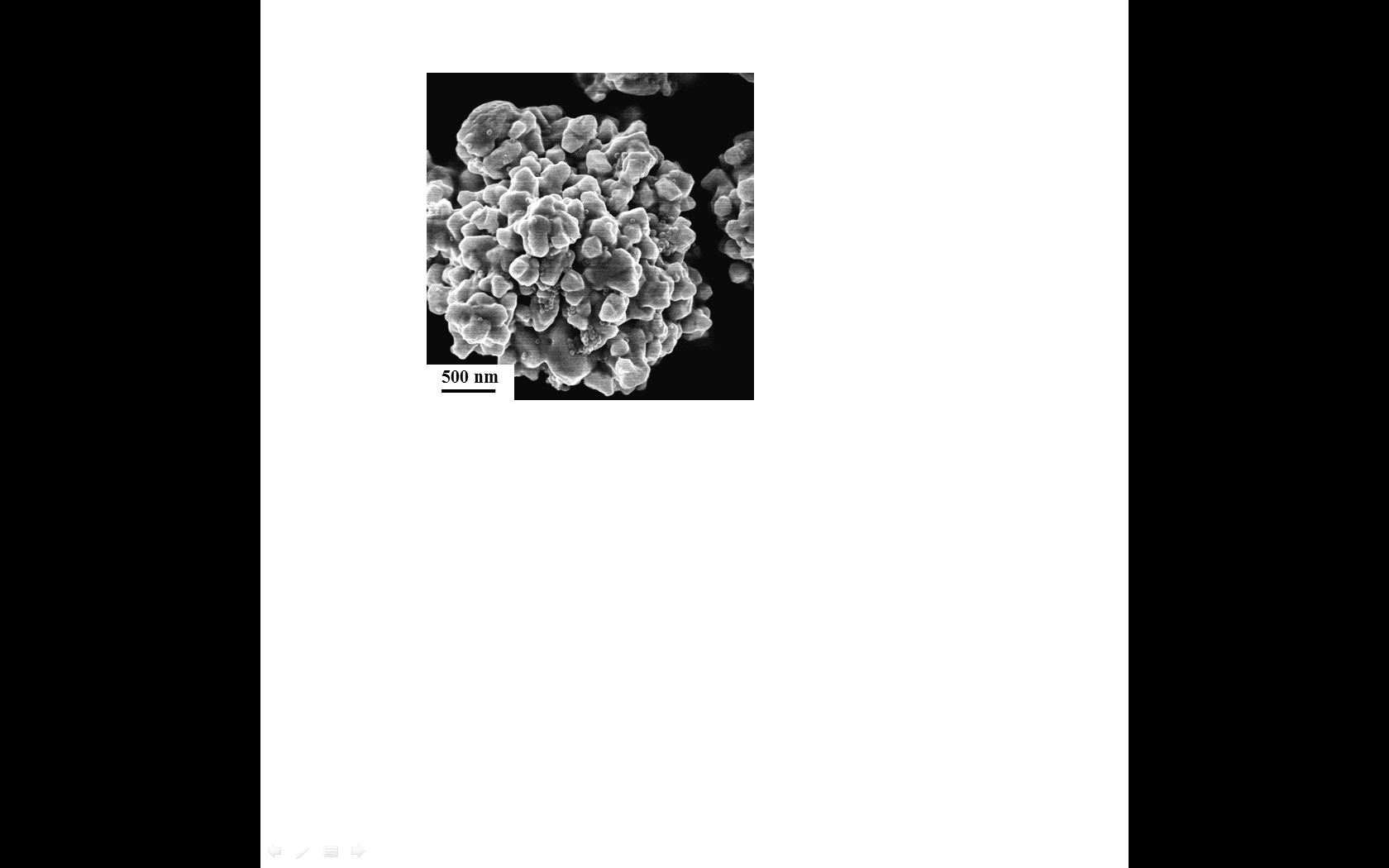


Supporting Figure 6. SEM image of particles synthesized without using a resin.

The products can be affected by the spacing between the silica NPs, as shown below. When a high concentration of amine functionalized NPs was treated to the beads, a relatively large number of NPs were immobilized on the bead, as shown in supporting Fig 7a. When we added gold source with base, gold was able to cover every NP, as shown in supporting Fig 7b. So in addition to the polydispersity, the shape can be affected.


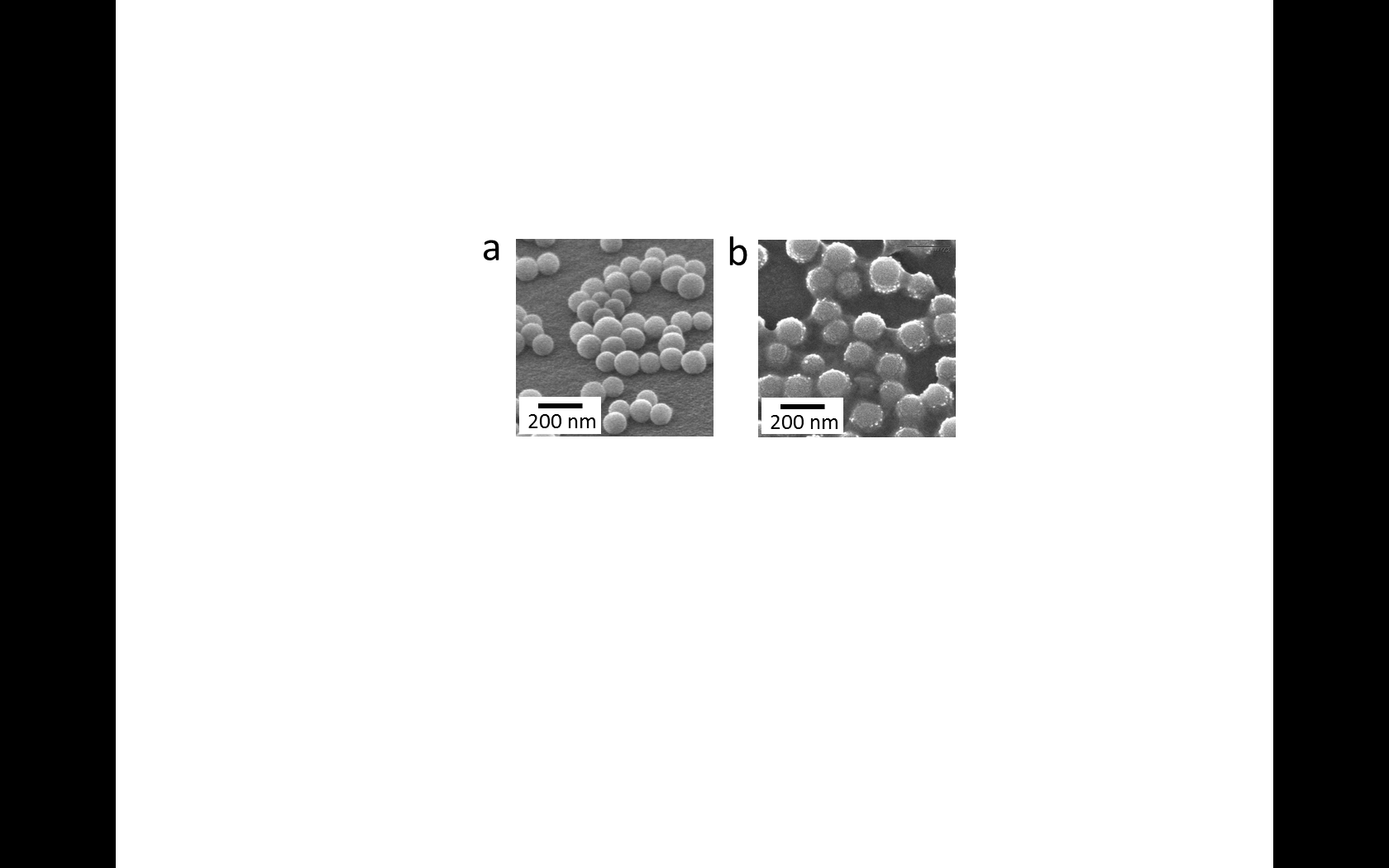


Supporting Figure 7. a) amine functionalized silica NPs immobilized bead, b) Au treated amine functionalized silica NPs immobilized bead.

To overcome the problem, we considered that the spacing between silica NPs on the beads could differ. We used 1) thiol-functionalized NPs which can have a charge-charge repulsion between NPs and 2) lower concentration of silica NPs to allow the presence of gaps. As a result, we could collect relatively less polydispersed particles (about 10% standard deviation)


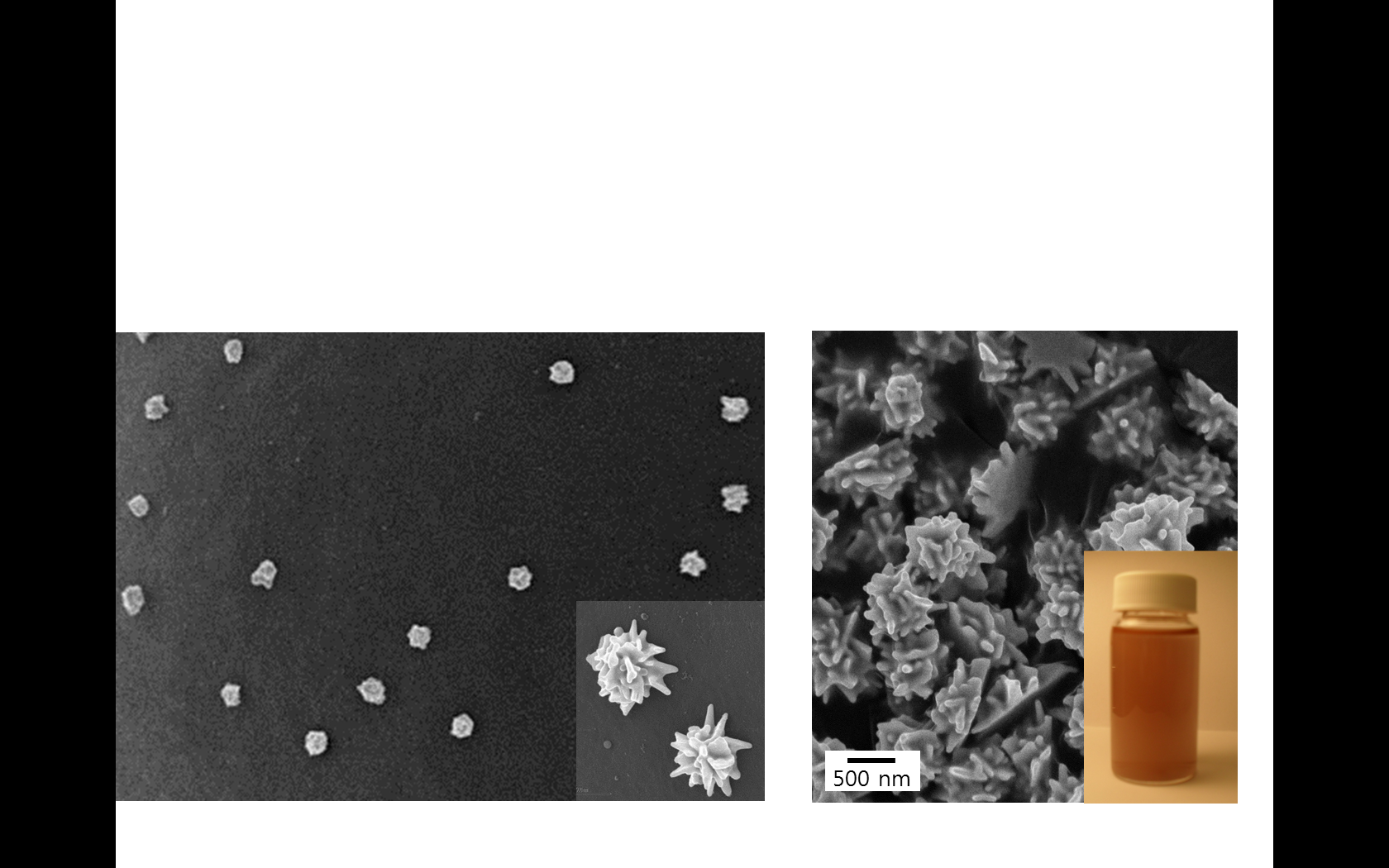


Supporting Figure 8. SEM image of 10 mg scale of nanoflower (box insert) corresponding photo image.

Supporting info 1. Calculation of Surface Area of 1 gram of beads.

Assumptions; Density = 1.05 g/cm^3^, Average diameter = 72 um

Therefore, the volume of 1 gram beads is 8.3 x 10^-2^ m^3^.

So, the volume of each bead is 2.0 x 10^-13^ m^3^.

The number of beads can be calculated from the total volume as 4.2 x 10^11^ beads.

Each individual bead has a surface area of 1.6 x 10^-8^ m^2^.

Therefore the surface area of 1 gram of beads is 6.7 x 10^3^ m^2^.


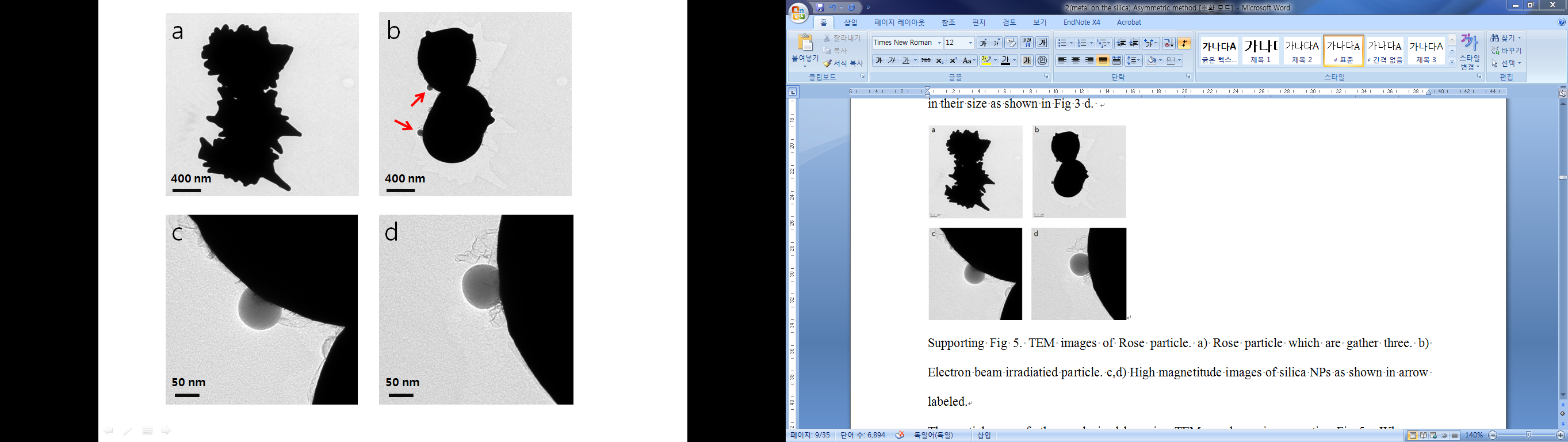


Supporting Fig 9. TEM images of nanorose particles. a) TEM image of three nanorose particles. b) Electron-beam-irradiated particles. c,d) High magnification images of silica NPs as indicated by the red arrows.


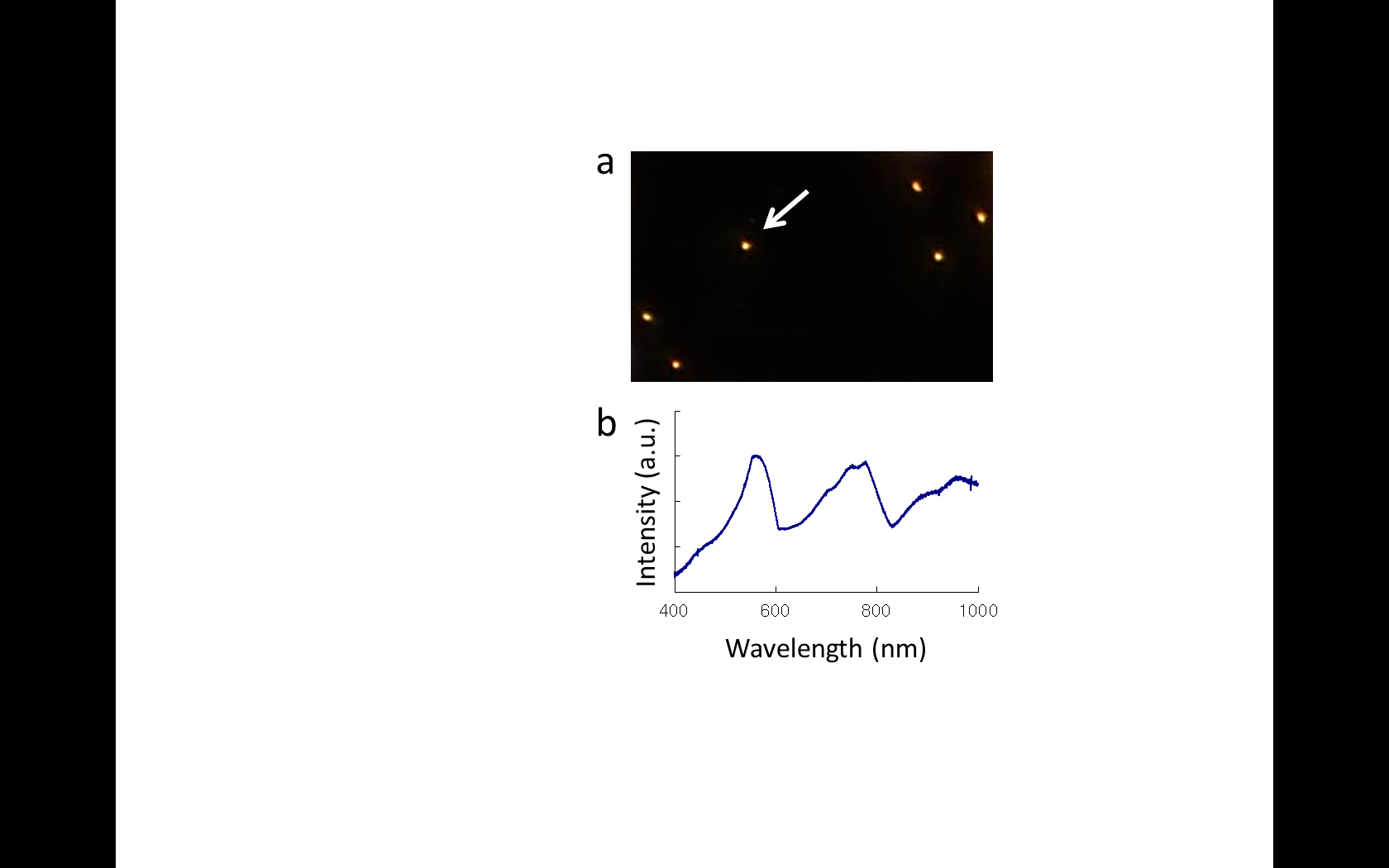


Supporting Figure 11. a) Dark-field image of rose-shape particles and b) Light scattering from corresponding rose-shape particle. They exhibit several peaks at 560 nm, 750, 780 nm and 965 nm.

Reference

1) Stober, W., A. Fink and E. Bohn *Journal of Colloid and Interface Science* 1968, 26**,** 62-&.

2) Petit, C., Lixon, P., and Pileni, M. *Journal of Physical Chemistry* 1993, 97, 49, 12974-12983.

3) Pileni, M. *Nature Materials* 2003, 2, 145-150.
